# Tat inhibition by didehydro-Cortistatin A promotes heterochromatin formation at the HIV-1 long terminal repeat

**DOI:** 10.1186/s13072-019-0267-8

**Published:** 2019-04-16

**Authors:** Chuan Li, Guillaume Mousseau, Susana T. Valente

**Affiliations:** Department of Immunology and Microbiology, The Scripps Research Institute, Jupiter, FL USA

## Abstract

**Background:**

Transcription from the integrated HIV-1 promoter is directly governed by its chromatin environment, and the nucleosome-1 downstream from the transcription start site directly impedes transcription from the HIV-1 promoter. The HIV-1 Tat protein regulates the passage from viral latency to active transcription by binding to the viral mRNA hairpin (TAR) and recruiting transcriptional factors to promote transcriptional elongation. The Tat inhibitor didehydro-Cortistatin A (dCA) inhibits transcription and overtime, the lack of low-grade transcriptional events, triggers epigenetic changes at the latent loci that “lock” HIV transcription in a latent state.

**Results:**

Here we investigated those epigenetic changes using multiple cell line models of HIV-1 latency and active transcription. We demonstrated that dCA treatment does not alter the classic nucleosome positioning at the HIV-1 promoter, but promotes tighter nucleosome/DNA association correlating with increased deacetylated H3 occupancy at nucleosome-1. Recruitment of the SWI/SNF chromatin remodeling complex PBAF, necessary for Tat-mediated transactivation, is also inhibited, while recruitment of the repressive BAF complex is enhanced. These results were supported by loss of RNA polymerase II recruitment on the HIV genome, even during strong stimulation with latency-reversing agents. No epigenetic changes were detected in cell line models of latency with Tat-TAR incompetent proviruses confirming the specificity of dCA for Tat.

**Conclusions:**

We characterized the dCA-mediated epigenetic signature on the HIV genome, which translates into potent blocking effects on HIV expression, further strengthening the potential of Tat inhibitors in “block-and-lock” functional cure approaches.

**Electronic supplementary material:**

The online version of this article (10.1186/s13072-019-0267-8) contains supplementary material, which is available to authorized users.

## Introduction

Current antiretroviral therapy (ART) effectively blocks HIV-1 replication and controls viremia in successfully treated patients; however, the integrated virus persists in a latent form primarily in resting memory T cells [1–3]. HIV-1 transcription is driven by the 5′ long terminal repeat (LTR), which is a strong promoter with multiple binding sites for cellular transcription factors. However, viral expression is almost silent once it is integrated into the host genome [4–6], as the host epigenetic machinery is well known to participate in transcriptional repression of HIV [7–10]. A specific array of three nucleosomes (Nuc), Nuc-0, Nuc-1 and Nuc-2, separated by DNase hypersensitive regions 1 and 2 (DHS-1, 2) are positioned at the HIV-1 promoter independently of integration site [11–13]. Nuc-1 is particularly important, it localizes immediately downstream of the transcription start site (TSS) and blocks the release of the promoter-proximal transcription complex [13, 14]. Nuc-1 plays a crucial role in the regulation of HIV transcription and contributes to viral silencing by serving as a target for epigenetic modifications that change the accessibility of the DNA sequence embedded inside. There are two types of epigenetic modifications: (1) ATP-dependent chromatin remodeling complexes which either move, eject or restructure nucleosomes; and (2) covalent histone modifications by specific enzymes, e.g., histone acetyltransferases (HATs), deacetylases, methyltransferase and kinases, that alter histone affinity for DNA [15]. Nuc-1 is regulated by chromatin remodeling complexes, the BAF (BRG1- or HBRM-associated factors) and PBAF (polybromo-associated factor) complexes. BAF and PBAF are human analogs of SWI/SNF (switching-defective-sucrose non-fermenting), an important family of proteins recruited to the HIV-1 promoter [16–18]. These complexes have opposite roles in Nuc-1 remodeling and HIV transcriptional regulation. BAF represses HIV transcription and helps the establishment of latency by positioning Nuc-1 downstream the TSS of HIV, while PBAF acts as a co-factor for Tat transactivation. Briefly, BAF, likely recruited by the short isoform of BRD4 [19], pulls Nuc-1 onto upstream DNA sequences less favorable for nucleosome formation immediately downstream of the TSS, leading to transcriptional repression [20–22]. Upon activation, BAF dissociates from the LTR, resulting in re-position of the nucleosomes to thermodynamically more favorable positions leading to de-repression of HIV transcription. Then the PBAF complex is recruited by the viral protein Tat, which re-positions nucleosomes downstream of TSS, enabling efficient transcriptional elongation [17, 18, 23, 24].

Covalent histone modifications at Nuc-1 such as deacetylation and methylations also regulate HIV transcriptional activity. Histone deacetylation is associated with heterochromatin and is mediated by histone deacetylases (HDACs), which can be recruited to the HIV promoter by transcription factors such as YY1 and CBF-1 to remove the acetyl groups from histones, resulting in viral latency [25–30]. Viral reactivation, on the other hand, is associated with histone acetylation by histone acetyltransferase (HATs) such as p300/CBP, PCAF and GCN5 which are recruited by host cell transcription factors and/or Tat [31–36]. In addition, histone methyltransferases (HMTs), such as SUV39H1, G9a and EZH2, deposit methyl groups to specific histone lysine sites that contribute to viral latency [37–40]. Thus, epigenetics impose reversible restrictions to the chromatinized provirus that may be overcome or reinforced by epigenetic drugs [41]. Drugs that inhibit histone deacetylation or methylation are potent latency-reversing agents (LRAs) [42], and on the other hand, drugs that block the cellular transcriptional machinery or reinforce deacetylation or methylation of histones lock HIV-1 into latency.

The early HIV-encoded Tat protein is required for robust transcription of the integrated viral genome by RNA polymerase II (RNAP II) [43, 44]. Tat binds to the 5′ terminal region of HIV nascent transcript’s stem-bulge-loop structure transactivation response element (TAR) and recruits the positive transcription elongation complex (P-TEFb) composed of cellular CDK9 and cyclin T1, to promote full-length viral transcripts [45–47]. Tat drastically enhances HIV transcription and is determinant in the transition between the latent to active state [48–51]. Unfortunately to date, there are no drugs against Tat in the clinic. We identified didehydro-Cortistatin A (dCA) as a potent and selective Tat inhibitor. dCA binds to the TAR-binding domain of Tat (known as “Arginine-rich motif,” ARM) inhibiting its interaction with TAR and preventing Tat transactivation of the HIV-1 promoter [52]. Treatment with dCA of primary CD4^+^ T cells isolated from infected individuals progressively blocks HIV-1 transcription, eventually driving viral expression into a state of persistent latency, refractory to reactivation by LRAs [53]. Importantly, in the bone marrow–liver–thymus (BLT) mouse model of HIV latency and persistence, adding dCA to ART-suppressed mice reduces viral RNA in tissues and significantly delays and diminishes viral rebound upon treatment interruption [54]. Together these results suggest that Tat inhibition by dCA progressively promotes heterochromatinization of the HIV promoter to lock HIV-1 into deep latency.

Here we characterized the epigenetic profile of the HIV promoter upon long-term treatment with dCA using four different HIV cell models with different transcriptional strengths: HeLa-CD4 cells chronically infected cells with NL4-3, were used as our model of high transcriptional activity; the promyelocytic OM-10.1 cell line [55–57] was used as a latent model with low transcriptional activity; U1 cell line containing proviruses with mutations in Tat [58, 59] was used as our model of suboptimal Tat activity; and finally ACH-2 cells with mutations in TAR [58, 60], and unresponsive to Tat, as our Tat transcriptional null model. Micrococcal nuclease (MNase) nucleosomal protection assays coupled with chromatin immunoprecipitations (ChIP) showed that long-term treatment with dCA does not alter the classic nucleosome positioning at the HIV-1 promoter; however, it promotes tighter nucleosome/DNA association that correlates with increased deacetylated histone 3 (H3) occupancy at Nuc-1. This repressive chromatin structure of the latent HIV-1 was consistent with loss of RNAP II and PBAF complex recruitment to the HIV genome and with an increase in the repressive BAF complex. This dCA-mediated epigenetic signature of the HIV genome that translates into potent blocking effects on HIV expression strengthens the potential of Tat inhibitors in “block-and-lock” HIV cure approaches.

## Methods

### Cell line and cell culture

HeLa-CD4 was provided by Uriel Hazan (Université de Cachan, France). Chronically infected HeLa-CD4 cells were obtained by infecting naïve cells with the NL4-3 virus, and passaging cells until the levels integrated HIV DNA level is stable. Cells were then maintained in the following ART cocktail: 200 nM Lamivudine, 200 nM Raltegravir, 100 nM Efavirenz (NIH AIDS Reagent Program, Division of AIDS, NIAID, NIH). HeLa-CD4 cells were cultured in DMEM (Thermofisher cat # 11965084) supplemented with 5% fetal bovine serum (FBS, Thermofisher cat # 10437028) and PSG (Thermofisher cat # 10378016) (penicillin 100 units/mL, streptomycin 100 μg/mL, l-glutamine 2 mM). OM-10.1 cells, U1 cells and ACH-2 cells were obtained from the NIH AIDS Reagent Program and maintained in RPMI 1640 media (Thermofisher cat # 11875093) supplemented with 10% FBS and PSG, in the presence of ART. For viral reactivation, cells were treated with SAHA (2.5 μM, LC Laboratories cat # V-8477), or PMA (20 nM, Fisher cat # BP6851) and Ionomycin (2 µM, Sigma # I3909) for 24 h in the presence of ART with or without dCA (10-100 nM).

### p24 ELISA

Quantification of HIV p24 capsid production was performed using the antigen capture assay kit from Advanced BioScience Laboratories, Inc. (cat # 5447), according to manufacturer’s protocol

### RT-PCR analysis of cell-associated RNA

Total RNA was extracted using RNeasy kit (Qiagen # 74106). First-strand cDNA was prepared from mRNA using random primers and the SuperScript III kit (Life Technologies cat #11752050). RT-PCR was performed using the LightCycler 480 SYBR Green I master system (Roche). The analysis was performed in triplicate, and the mean RNA expression level normalized to GAPDH mRNA. HeLa-CD4 cells, OM-10.1 cells and ACH2 cells mRNA were analyzed with primers to Nef region, and U1 cells mRNA analyzed with primers to Vpr region (Additional file 1: Table S1).

### Quantification of integration events

Genomic DNA was prepared using the DNeasy blood and tissue kit (Qiagen cat # 69506). Integration events were quantified by Alu-Gag PCR, followed by nested RT-PCR with primers to the gag region (Additional file 1: Table S1) as previously described [53].

### Micrococcal MNase protection assay

Nucleosome position was monitored using methods by Jadlowsky and Rafati with some modifications [24, 54, 61]. Briefly, cells were cross-linked similarly to ChIP protocol below, then washed with buffer B (0.25% Triton-X 100, 1 mM EDTA, 0.5 mM EGTA, 20 mM Hepes, pH 7.6), buffer C (150 mM NaCl, 1 mM EDTA, 0.5 mM EGTA, 20 mM Hepes, pH 7.6). After one wash in cold PBS, 1.5 × 10^7^ cross-linked cells were re-suspended in 1 mL hypotonic buffer A (300 mM sucrose, 2 mM Mg Acetate, 3 mM CaCl_2_, 10 mM Tris–HCl pH 8.0, 0.1% Triton-X 100, 0.5 mM DTT), incubated on ice for 5 min, and dounced 20 times with 2 mL homogenizer (tight pestle, Wheaton). Nuclei were collected by centrifuging at 720×*g* for 5 min at 4 °C. Pellets were re-suspended in 1 mL buffer D (25% glycerol, 5 mM Mg acetate, 50 mM Tris–HCl pH 8.0, 0.1 mM EDTA, 5 mM DTT) at 1.5 × 10^7^ nuclei/mL. The pellets were collected by centrifuging at 4 °C for 5 min at 720×*g*. The pellets were re-suspended in 1 mL buffer MN (60 mM KCl, 15 mM NaCl, 15 mM Tris–HCl pH 7.4, 0.5 mM DTT, 0.25 mM sucrose, 1 mM CaCl_2_) at 2.5 × 10^7^ nuclei/mL. The equivalent of 2.5 × 10^6^ nuclei was used per digestion reaction. Micrococcal nuclease (Fisher Scientific cat # 50645000) diluted in buffer MN was added so that 25 (Digested) and 0.25 (Undigested) total units were used per 100 μL reaction and digested for 30 min at room temperature. Reactions were stopped with 12.5 mM EDTA and 0.5% SDS. After 4 h of proteinase K digestion at 37 °C, each sample was de-cross-linked overnight at 65 °C in the presence of 200 mM NaCl. The products were digested with RNase for 30 min and purified with PCR cleanup kit (Qiagen cat # 28106). Eluted products were run on a 2% agarose gel to confirm MNase digestion. After measuring DNA concentration, samples were diluted to 5 ng/μL and used for real-time PCR (RT-PCR) analysis (Bio-rad, CFX96™ Real-Time System). Primers are listed in supplemental (Additional file 1: Table S1). The fold difference was calculated using the delta *C*_*T*_ method between digested and undigested samples.

### Chromatin immunoprecipitation assay

The ChIP assay was performed as previously described with some modifications [52–54]. Cells were cross-linked with 1% formaldehyde for 10 min and quenched with 0.125 M glycine for 5 min at room temperature. Pellets of 1 × 10^7^ cells were sonicated 18 times for 10-s bursts on ice to generate sheared chromatin of 200 to 400 nucleotides. The protein concentration in the sonicated sample was quantified with the Bradford protein assay (Bio-Rad cat # 5000006). A total of 500 μg protein was used for each IP with antibody anti RNAP II (Millipore cat # 05-623), BAF180 (Millipore cat # ABE70), BAF250 (Millipore cat # 04-080), H3 (Millipore cat # 07-690), acetylated H3K27 (Millipore cat # 07-517-683), or controls, normal mouse IgG (Millipore cat # NI03) and rabbit IgG (Fisher Scientific cat # NB810569101). The equivalent of 1% chromatin was saved as input control. Immunoprecipitated DNA was eluted with buffer (0.1 M NaHCO3, 1% SDS) at 30 °C for 15 min. DNA samples were treated with RNase A (Thermo Scientific cat # FEREN0531) for 30 min at 37 °C, reverse cross-linked for 4 h at 65 °C in the presence of 200 mM NaCl, and then digested with proteinase K (Fisher Scientific cat # BP1700100) at 60 °C for 1 h. The DNA was purified using a PCR clean kit (Qiagen cat # 28106). Primers used are listed in Additional file 1: Table S1. Input (1%) was used to standardize results. The relative proportions of coimmuno-precipitated DNA fragments were determined on the basis of the threshold cycle (*C*_*T*_) for each RT-PCR product. The data sets were normalized to input values (percent input = 2[*C*_T_ Input − *C*_T_ IP] × 100). The average value of the IgG background for each primer was subtracted from the raw data.

### Western blot analysis

Cells were lysed in RIPA buffer supplemented with complete EDTA-free protease inhibitor cocktail (Roche cat # 04293132001), and the lysate centrifuged at 12,000 rpm for 10 min at 4 °C. The protein concentration in the supernatant was quantified with the Bradford protein assay (Bio-Rad cat # 5000006). Total protein extracts were separated by SDS-PAGE and transferred onto polyvinylidene difluoride membranes. Membranes were probed with anti-H3 (Millipore cat # 07-690) and anti-acetylated H3 (Millipore cat # 06-599), followed by horseradish peroxidase-conjugated anti-rabbit IgG goat polyclonal antibody (Sigma cat # A0545).

### Statistical analysis

P-values for in vitro experiments were calculated using one-way ANOVA and Kruskal–Wallis with post hoc Dunn multiple comparison analysis or unpaired t test with 95% confidence intervals using the Prism 7 for Macintosh (GraphPad Software, La Jolla, CA).

## Results

### dCA blocks HIV expression in chronic infected HeLa-CD4 cells

**Fig. 1:**
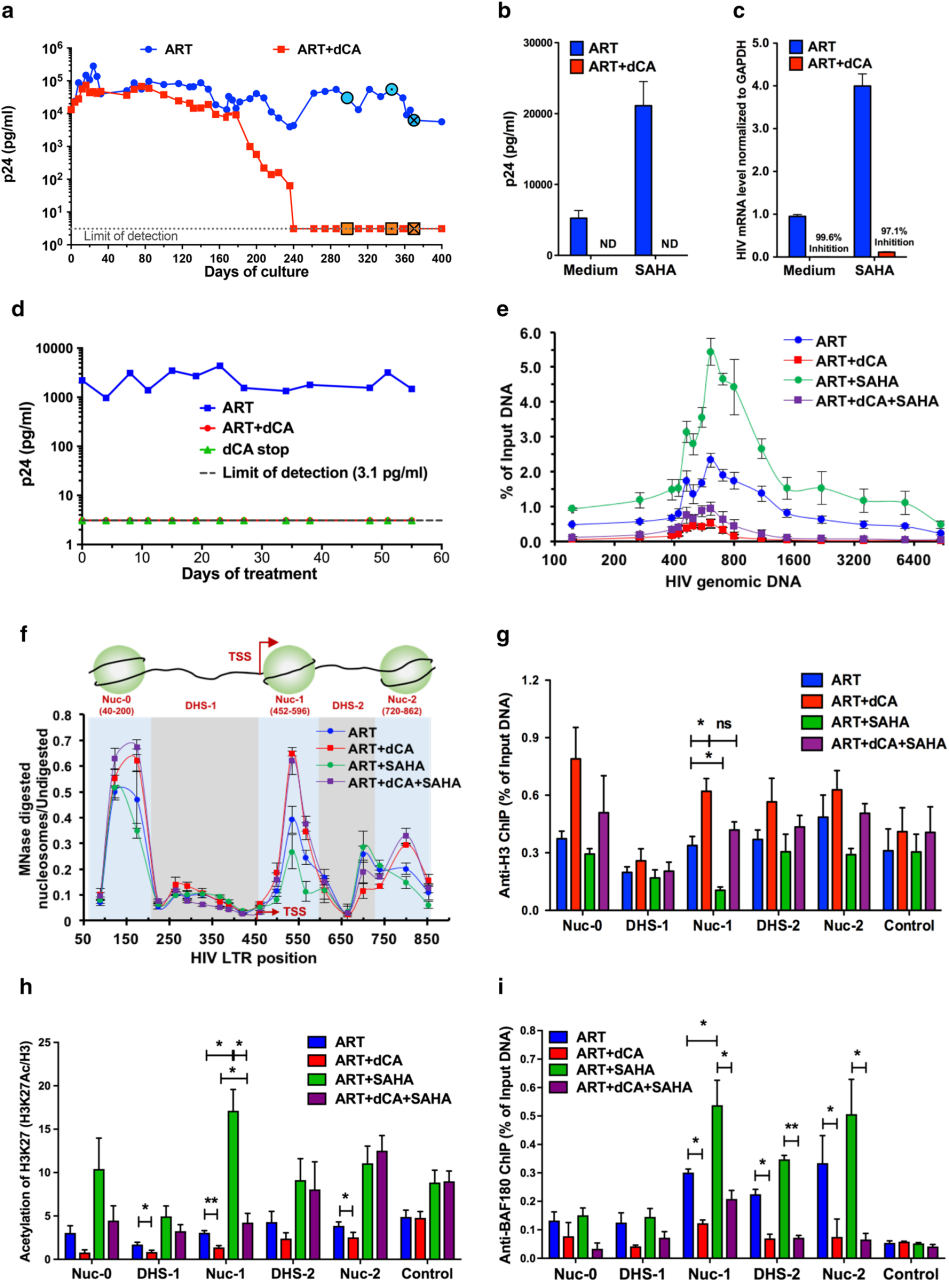
dCA promotes a repressive chromatin environment at the HIV promoter in HeLa-CD4 chronically infected cells. **a** dCA reduces viral transcription overtime in NL4-3 chronically infected HeLa-CD4 cells. Cells were split on average every 3 days in the presence of ART with or without 10 nM dCA. Capsid production in the supernatant was quantified via p24 ELISA. Data are representative of three independent experiments. **b** SAHA treatment activated viral production in chronically infected HeLa-CD4 cells. After day 280 of culture, cells (highlighted with “⦿/☒” in panel A) treated with ART and ART + dCA were stimulated with 2.5 µM SAHA for 24 h. Capsid production in the supernatant was quantified by a p24 ELISA. Data are average of 3 independent experiments, error bars represent standard deviation (SD) of 3 independent experiments (ND, not detected). **c** SAHA treatment increased viral mRNA levels in chronically infected HeLa-CD4 cells. Cell samples from panel B were also used for RNA extraction, and cDNAs from extracted total RNA were quantified by RT-PCR using primers to the Nef region. Results were normalized as the number of viral mRNA copies per GAPDH mRNA. Viral mRNA generated in the ART control was set to 100%, error bars represent SD of 3 independent experiments. **d** Viral production in HeLa-CD4 cell line upon treatment interruption. Treatment of dCA was stopped on day 360 and the cells were further maintained in ART for another 55 days. Capsid production was quantified via p24 ELISA. Data are representative of three independent experiments. **e** Distribution of RNAP II on HIV genome DNA. ChIP assay to RNAP II was performed using cells samples from panels B-C. Results are represented as the percentage of input and subtracted the background of the isotype IgG control. Data are average of 3 independent experiments, and error bars represent SD of 3 experiments for each primer set. **f** Chromatin structure of the HIV LTR upon dCA treatment was determined by MNase protective assays. The amount of the MNase digested PCR product was normalized to that of the undigested product using the ∆C(t) method (*y*-axis), which is plotted against the midpoint of the corresponding PCR amplicon (*x*-axis). The *X*-axis represents base pairs units with 0 as the start of HIV LTR. Error bars represent the SD of 3 independent experiments. **g** H3 occupancy on HIV promoter DNA determined by ChIP to H3. Results are presented as percent immunoprecipitated DNA over input, after background subtraction of the isotype IgG control. The ORF of GAPDH was used as control. Data are average of 3 independent experiments and error bars represent the SD of 3 experiments for each primer set. **h** The level of H3 lysine 27 acetylation (H3K27Ac) on HIV promoter DNA determined by H3K27Ac ChIP. The results are presented as acetylation of H3K27 over total Histone H3 level, after subtraction of the isotype IgG control background. The promoter of RPL-10 was used as the control. Data are average of 3 independent experiments and error bars represent the SD of 3 experiments for each primer set. **i** The recruitment of PBAF complex on HIV promoter DNA determined by ChIP to BAF180. Results are presented as percent immunoprecipitated DNA over input, after isotype IgG control background subtraction. The promoter of GAPDH was used as the control. Data are average of 3 independent experiments and error bars represent SD of 3 experiments for each primer set. Statistical significance was determined using unpaired t-test (ns, no significance, *P < 0.05, **P < 0.01)

To investigate the long-term epigenetic activity of dCA on highly active HIV promoter, HeLa-CD4 cells chronically infected cells with NL43, a mixture of multiply infected cells with various HIV integration sites, were used as our model of high transcriptional activity. Infected HeLa-CD4 cells were maintained with an ART cocktail [Lamivudine (200 nM), Raltegravir (200 nM), Efavirenz (100 nM)] with or without 10 nM of dCA, and split every 3–4 days while capsid production in the supernatant was measured by p24 ELISA (Fig. 1a). ART treatment alone maintained viral production at approximately 10^5^ pg/mL, while ART + dCA treatment continuously reduced viral production over time to undetectable levels by day 240. To ensure the inhibition of HIV capsid production upon ART + dCA treatment was not due to an enrichment of uninfected HeLa-CD4 cells, we determined the level of integrated proviruses by Alu-PCR, using cell samples at 80, 120, 280 and 360 days of culture (Additional file 2: Fig. S1A). Similar or higher levels of integrated HIV DNA were observed in dCA-treated cells, supporting transcriptional inhibition by dCA. The increase in proviral integration in ART + dCA-treated cells observed after day 280 is likely due to a survival advantage of cells with multiply integrated proviruses, as opposed to infected cells in ART-only-treated cells which may be counter selected from cytotoxic viral protein expression. Next, we ensured these cells grown for a long period of time were still responsive to various LRA stimuli. We analyzed thioredoxin-binding protein-2 (TBP-2) and interleukin 1ß (IL-1ß) mRNA production, upon treatment with the HDAC inhibitor suberoylanilide hydroxamic acid (SAHA) and PMA/ionomycin, respectively [62–64]. No differences in TBP-2 and IL-1ß upregulation by SAHA or PMA/Ionomycin were observed with either long-term treatment (280 days) as compared to uninfected HeLa-CD4 cells (Additional file 2: Fig. S1b, c). Furthermore, a western blot to N-terminus acetylated H3 (Ac-H3) revealed similar Ac-H3 upregulation by SAHA in both ART- and ART + dCA-treated cells (Additional file 2: Fig. S1d), indicating that these long-term treatments had not altered cell viability and responsiveness to activating stimuli. We then investigated whether cells treated with dCA could be reactivated by different stimulators. We treated cells (at the 280-day time point) with SAHA or PMA/ionomycin for 24 h in the presence of ART or ART + dCA. The capsid production was assayed by p24 ELISA, and cell-associated mRNA was measured by RT-PCR with primers to the Nef region. When stimulated with SAHA, ART-treated cells showed about a fourfold increase in capsid production, while in ART + dCA-treated cells viral production remained below detection (Fig. 1b). Similarly, the RT-PCR analysis revealed that viral mRNA expression in ART + dCA-treated cells was drastically blocked in both unstimulated cells (99.6%) and stimulated (97.1%) conditions (Fig. 1c). We observed similar results when cells were stimulated with PMA/ionomycin (Additional file 2: Fig. S1e and f); however, SAHA in these cells was a better reactivator of both p24 level and HIV-1 mRNA levels.

Supporting these results, on day 400, treatment with dCA was interrupted and no viral rebound was observed during the next 55 days (Fig. 1d), confirming dCA’s long-lasting inhibitory effect on HIV transcription [53]. Using this now characterized model of dCA-mediated “deep-latency,” refractory to viral reactivation, we investigated the epigenetic profile at the HIV-1 promoter and genome. ChIP to RNAP II followed by RT-PCR revealed that in cells maintained on ART, RNAP II accumulated in the promoter-proximal region and was detected throughout the entire HIV genome (Fig. 1e, blue line), consistent with poised promoters [65]. Reactivation with SAHA increased overall recruitment of RNAP II at the promoter and throughout the genome (Fig. 1e, green line), paralleling increased capsid expression (Fig. 1b) and viral mRNA productions (Fig. 1c). In ART + dCA-treated cells (Fig. 1e, red line), a drastic decrease in RNAP II recruitment was observed in the promoter-proximal region and close to background levels in the distal genomic region, consistent with HIV transcription blocked by dCA (Fig. 1b and c). Upon treatment with SAHA, only a small increase in RNAP II recruitment was observed at the promoter region but not in distal genomic downstream regions (Fig. 1e, purple line), reflecting failed reactivation of the latent provirus (Fig. 1b and c). Similar results were obtained using PMA/ionomycin as the activator (Additional file 2: Fig. S1g). Taken together, these results suggest that dCA treatment promotes a chromatin environment at the HIV-1 promoter that is not amenable to RNAP II recruitment and transcriptional elongation.

### dCA reinforces the typical latent nucleosome organization on the HIV promoter DNA

Nucleosomes are precisely positioned in three distinct positions at the HIV promoter: Nuc-0 (400–200 nt), Nuc-1 (452–596 nt), and Nuc-2 (702–864 nt), and these regulate the promoter activity by directly limiting access of transcription factors to the promoter [11, 12, 66, 67]. To investigate the repressive chromatin environment at the HIV-1 promoter, we used MNase protective assays to study this nucleosomal organization after long-term treatment with dCA, on three independent time points after day 280, when viral transcripts were no longer detected in ART + dCA-treated cells. The collected mononucleosomes and isolated DNA was either digested or not with MNase, prior to PCR amplification with 20 individual but overlapping 100 bp amplicons covering the HIV LTR region [24, 54]. Results are plotted as a ratio of digested over undigested DNA quantification (Fig. 1f). In chronically infected HeLa-CD4 cells, we observed the three classical MNase-protected regions at the promoter region, which reflect Nuc-0 at the 5′ end of the promoter, Nuc-1 immediately downstream the TSS, and Nuc-2 at the 3′ end of the promoter, separated by DHS-1 and 2. The treatment with dCA (Fig. 1f, red line) increased MNase protection at Nuc-1 and Nuc-2, suggesting tighter nucleosome binding at these nucleosomes. When ART-treated cells were reactivated by SAHA, we observed loss of protection in Nuc-1 and Nuc-2 regions (Fig. 1f, green line), an expected outcome as histone acetylation weakens the interaction with DNA [68]. However, stimulation with SAHA failed to induce the rearrangement of nucleosome structure in ART + dCA-treated cells (Fig. 1f, purple line), consistent with the inhibitory effects on viral reactivation (Fig. 1b, c). Viral reactivation using PMA/ionomycin also failed to induce the nucleosome rearrangement in dCA-treated cells (Additional file 2: Fig. S1h). To ascertain the increased MNase protection at Nuc-1 region due to increased nucleosome occupancy during treatment with dCA, we performed ChIP to H3 (Fig. 1g). A significant increase in H3 at Nuc-1 region was apparent in ART + dCA compared to ART alone treated cells, and even if no statistical differences were observed at Nuc-0 and Nuc-2, H3 levels trended higher in ART + dCA versus ART-treated cells (Fig. 1g, *red versus blue bars*). In ART-treated cells, H3 occupancy at Nuc-1 decreased significantly upon stimulated with SAHA (Fig. 1g, *green bar*), consistent with chromatin relaxation. However, H3 occupancy at the Nuc-1 region in ART + dCA-treated samples after SAHA activation was not significantly reduced (Fig. 1g, *red versus purple bars*), and the absolute amount of H3 coverage remained high in ART + dCA-treated cells. Taken together, these results suggest dCA promotes the establishment of typical latent nucleosome positioning at the HIV promoter DNA; however, it is characterized by increased Nuc-1 occupancy downstream the TSS, rendering viral reactivation upon stimulation with LRAs much less likely to occur.

Histone modifications regulate the accessibility of chromatin DNA, and Nuc-1 acetylation at the HIV-1 promoter is associated with HIV reactivation [68]. We next performed ChIP against histone H3 lysine 27 (H3K27Ac), a marker of HIV LTR promoter activation and open chromatin [69], and normalized the results to the H3 level. As expected, SAHA treatment promoted increased H3K27 acetylation especially at Nuc-1 (Fig. 1h, *green bar*). dCA significantly decreased H3K27Ac levels primarily at Nuc-1 with or without SAHA (Fig. 1h, *red and purple bar*). Meanwhile, dCA did not alter the H3K27Ac levels at the RPL-10 promoter used as control, which is also sensitive to SAHA, confirming dCA specificity to Tat and thus HIV promoter chromatin.

BAF and PBAF complexes have opposite roles in HIV transcriptional regulation by re-positioning of nucleosomes [24, 70]. We performed ChIP to investigate whether dCA treatment changes the recruitment of BAF or PBAF complexes to the HIV LTR. These complexes share many proteins; however, PBAF specifically includes BAF180 which is excluded from BAF, while BAF includes BAF250 which is excluded from the PBAF complex. These differences allow us to distinguish the two complexes. ChIP against BAF180 (Fig. 1i) confirmed that PBAF is enriched at Nuc-1, DHS-2, and Nuc-2 compared to Nuc-0 and DHS-1 in ART-treated cells, and further enriched when cells were stimulated with SAHA or PMA/ionomycin, indicating transcriptional activation (Fig. 1i and Additional file 2: Fig. S1i). In the presence of dCA, BAF180 recruitment to Nuc-1, DHS-2, and Nuc-2 is decreased by more than half in both unstimulated and stimulated cells (Fig. 1i and Additional file 2: Fig. S1i). Since the binding of BAF is mutually exclusive of PBAF, we performed ChIP to BAF250. The values were too low when stimulating with SAHA, however, when using PMA/Ionomycin as an LRA, dCA increased BAF250 binding on Nuc-1, DHS-1, and Nuc-2 (Additional file 2: Fig. S1j). Collectively, these results demonstrate that inhibition of Tat by dCA results in an extremely repressive chromatin environment at the HIV-1 promoter, characterized by low H3K27 acetylation levels and little PBAF recruitment.

### dCA changes the chromatin signature of the HIV promoter DNA in latently infected cells

**Fig. 2:**
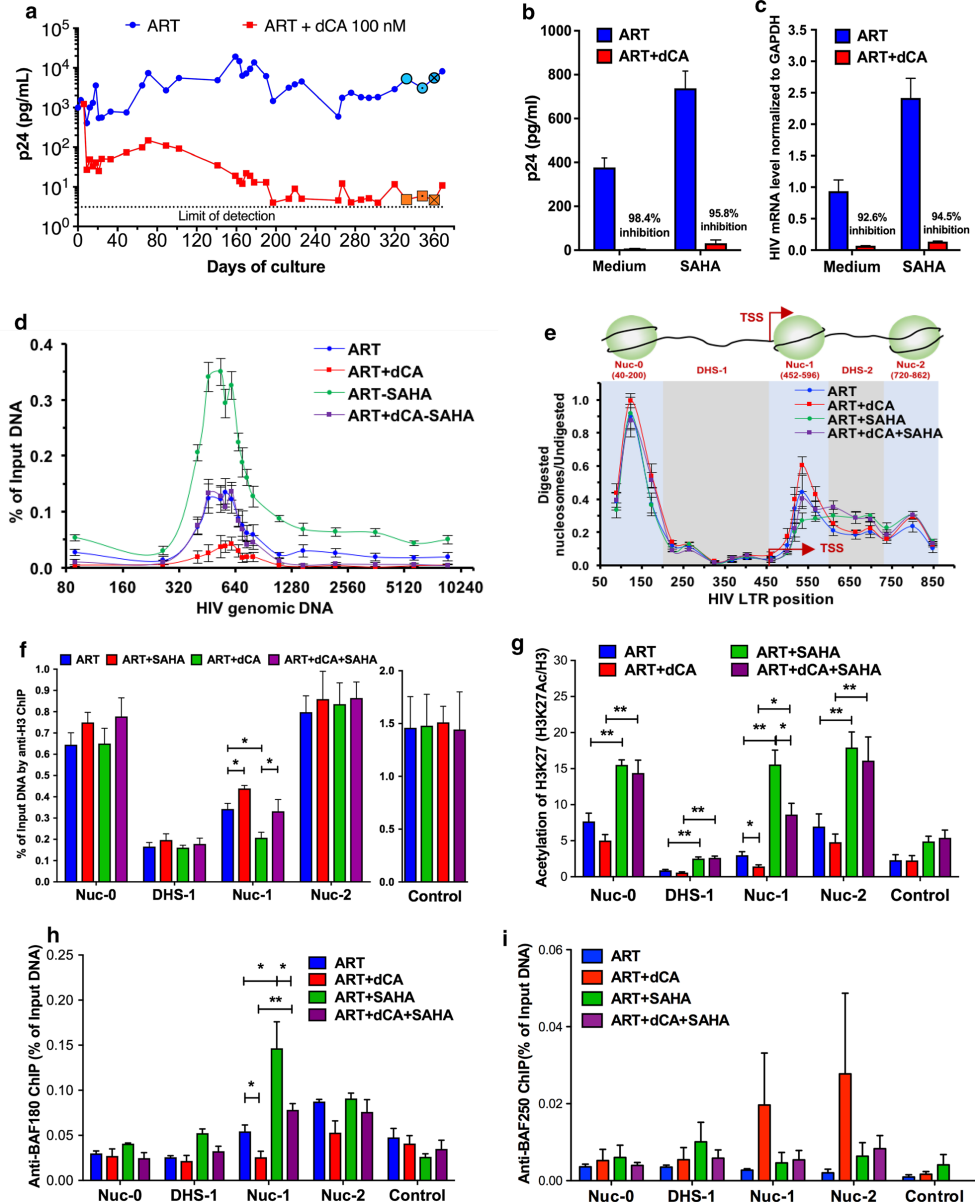
dCA induces a repressive chromatin environment on the HIV promoter in latently infected OM-10.1 cells. **a** Viral production in HIV latently infected OM-10.1 cell treated with ART with or without 100 nM dCA. Cells were split and treated on average every 3 days and capsid production in the supernatant was quantified by p24 ELISA. Data are representative of three independent experiments. **b** SAHA induced viral production in OM-10.1 cells. After 300 days treatment with ART and ART + dCA, cells (highlighted with “⦿/☒” in panel A) were stimulated with 2.5 µM SAHA for 24 h. Capsid production was quantified via a p24 ELISA. Data are average of 3 independent experiments, and the error bars represent SD of 3 independent. **c** SAHA induced viral mRNA level in OM-10.1 cell. Cellular sample from panel B also used for RNA extraction, and cDNAs from extracted total RNA were quantified by RT-PCR using primers to the Nef region. Results were normalized as the number of viral mRNA copies per GAPDH mRNA. Viral mRNA generated in the ART control was set to 100%, and the error bars represent the SD of 3 independent experiments. **d** Distribution of RNAP II on HIV genome DNA. ChIP assay to RNAP II was performed using cells samples from panels B-C. Results are represented as the percentage of input and subtracted the background of the isotype IgG control. Data are average of 3 independent experiments and error bars represent SD of 3 experiments for each primer set. **e** The chromatin structure of the HIV LTR in latent OM-10.1 cell from sample in panel B and C. **f** Histone H3 occupancy on HIV promoter DNA determined by ChIP. Results are represented as the percentage of input and subtracted the background of the isotype IgG control. The ORF of GAPDH was used as control. Data are average of 3 independent experiments, and error bars represent the SD of 3 experiments for each primer set. **g** The level of H3 lysine 27 acetylation (H3K27Ac) on HIV promoter DNA determined by H3K27Ac ChIP. The results are presented as acetylation of H3K27 over total Histone H3 level, after subtraction of the isotype IgG control background. The promoter of RPL-10 was used as the control. Data are average of 3 independent experiments, and error bars represent the SD of 3 experiments for each primer set. **h** The recruitment of PBAF complex on HIV promoter DNA determined by ChIP to BAF180. Results are presented as percent immunoprecipitated DNA over input, after isotype IgG control background subtraction. Data are average of 3 independent experiments, and error bars represent the SD of 3 experiments for each primer set. The promoter of GAPDH was used as the control. **i** Recruitment of BAF250 to the HIV promoter by ChIP. Results are presented as percent immunoprecipitated DNA over input, after isotype IgG control background subtraction. Data are average of 3 independent experiments, and error bars represent the SD of 3 experiments for each primer set. The promoter of GAPDH was used as the control. Statistical significance was determined using the unpaired t-test (*P < 0.05, **P < 0.01)

To investigate the long-term epigenetic activity of dCA on a model of low transcriptional activity, we used latently infected OM-10.1 cells. These cells were cloned from HL-60 promyelocyte cells that survived an acute HIV-1 infection and contain a single integrated provirus [55–57]. Previously we reported the ability of dCA to repress residual HIV transcription and production from the latent cell model OM-10.1 [53] (Fig. 2a), which is a clone derived from HL-60 promyelocytic cells that survived an acute HIV-1 NL4-3 infection [56]. In latent OM-10.1 cells, dCA inhibits residual viral production to almost undetectable levels, and dCA-mediated viral latency is refractory to reactivation by multiple LRAs, such as SAHA, TNF-α, or prostratin [53, 54]. This is accompanied by the loss of RNAP II recruitment to the viral promoter, as well as distal HIV genomic regions [54]. Here, to further investigate the long-lasting activity of dCA on a latent HIV infection, these cells were maintained in an ART cocktail with or without dCA, split every 3-4 days, and capsid production in the supernatant was measured via p24 ELISA (Fig. 2a). After day 300, when viral production was minimal, cells were stimulated with SAHA for 24 h in the presence of ART or ART + dCA. The viral production in ART + dCA-treated cells was drastically blocked in both unstimulated (98.4%) and stimulated (95.8%) cells (Fig. 2b). Similarly, the RT-PCR analysis revealed that viral mRNA expression in ART + dCA-treated cells was drastically inhibited in both unstimulated cells (99.6%) and stimulated (97.1%) conditions (Fig. 2c).

Next, ChIP to RNAP II using these samples revealed that dCA decreases RNAP II recruitment on the promoter DNA in both unstimulated and stimulated conditions (Fig. 2d). SAHA treatment promoted increased RNAP II recruitment to the promoter region as well as throughout the genomic region in ART-treated cells, paralleling the upregulation of transcription shown in panels B-C (Fig. 2d). In ART + dCA-treated cells however, SAHA promoted a smaller increase in RNAPII recruitment at the promoter region, but not in the distal downstream regions of the genome, consistent with the failed reactivation of the latent provirus observed in panels B-C (Fig. 2d).

MNase protection assays were used to investigate the nucleosomal structure of HIV promoter DNA in this model. In ART + dCA-treated cells, we observed increased Nuc-1 protection as compared to ART-treated cells in both unstimulated and stimulated conditions, suggesting a tighter nucleosome at Nuc-1 in dCA-treated cells (Fig. 2e). A reduction in MNase protection was observed when both treated cells were stimulated with SAHA; however, the occupancy at Nuc-1 in stimulated  ART + dCA-treated cells was still comparable with that in unstimulated ART-treated cells, suggesting that dCA decreases the chromatin accessibility at the promoter DNA by promoting its heterochromatinization. Next, ChIP to histone H3 was performed (Fig. 2f), and consistent with the MNase protection, ART + dCA cells show increased histone H3 occupancy at Nuc-1 compared with ART-treated cells in both unstimulated and stimulated conditions, suggesting increased nucleosome occupancy (Fig. 2f). Decrease in histone H3 occupancy was observed when cells were stimulated with SAHA; however, the histone H3 occupancy at Nuc-1 in ART + dCA-treated cells was comparable to that of unstimulated ART-treated cells, further strengthening that dCA increases the histone H3 occupancy at Nuc-1.

ChIP analysis of H3K27Ac normalized to H3 levels revealed a fairly high level of acetylation at all three nucleosomes under basal conditions in ART-treated cells (Fig. 2g, blue bars), consistent with ongoing detectable viremia (Fig. 2a), and a drastic increased in acetylation upon SAHA treatment (Fig. 2g, green bars). ART + dCA treatment significantly decreased H3K27Ac level at Nuc-1 in basal and SAHA activated conditions (Fig. 2g, red and purple bars). These results are consistent with accrued nucleosome and histone occupancy at Nuc-1 (Fig. 2e, f) [54]. The promoter of RPL-10 used as the control is sensitive to SAHA and thus, as expected, H3K27Ac was increased at the promoter, while not affected by dCA (Fig. 2g). ChIP to PBAF complex (BAF 180) revealed its presence at Nuc-1 and Nuc-2 of the HIV promoter and further enrichment at Nuc-1 upon stimulation with SAHA (Fig. 2h). In dCA-treated cells, however, BAF180 at Nuc-1 before and after SAHA treatment was significantly reduced (Fig. 2h). Since PBAF and BAF are mutually exclusive, it was not unexpected to observe higher recruitment of BAF250 at Nuc-1 and Nuc-2 in dCA-treated cells (Fig. 2i), which translates into a repressive chromatin environment at the HIV promoter fairly insensitive to SAHA. Taken together, these results confirm a repressive chromatin environment, characterized by reduced histone acetylation at Nuc-1, occluding RNAPII recruitment and transcriptional elongation in latently infected OM-10.1 cell after long-term treatment with dCA.

### dCA partially block HIV expression in U1 cells with suboptimal Tat-TAR activity

**Fig. 3:**
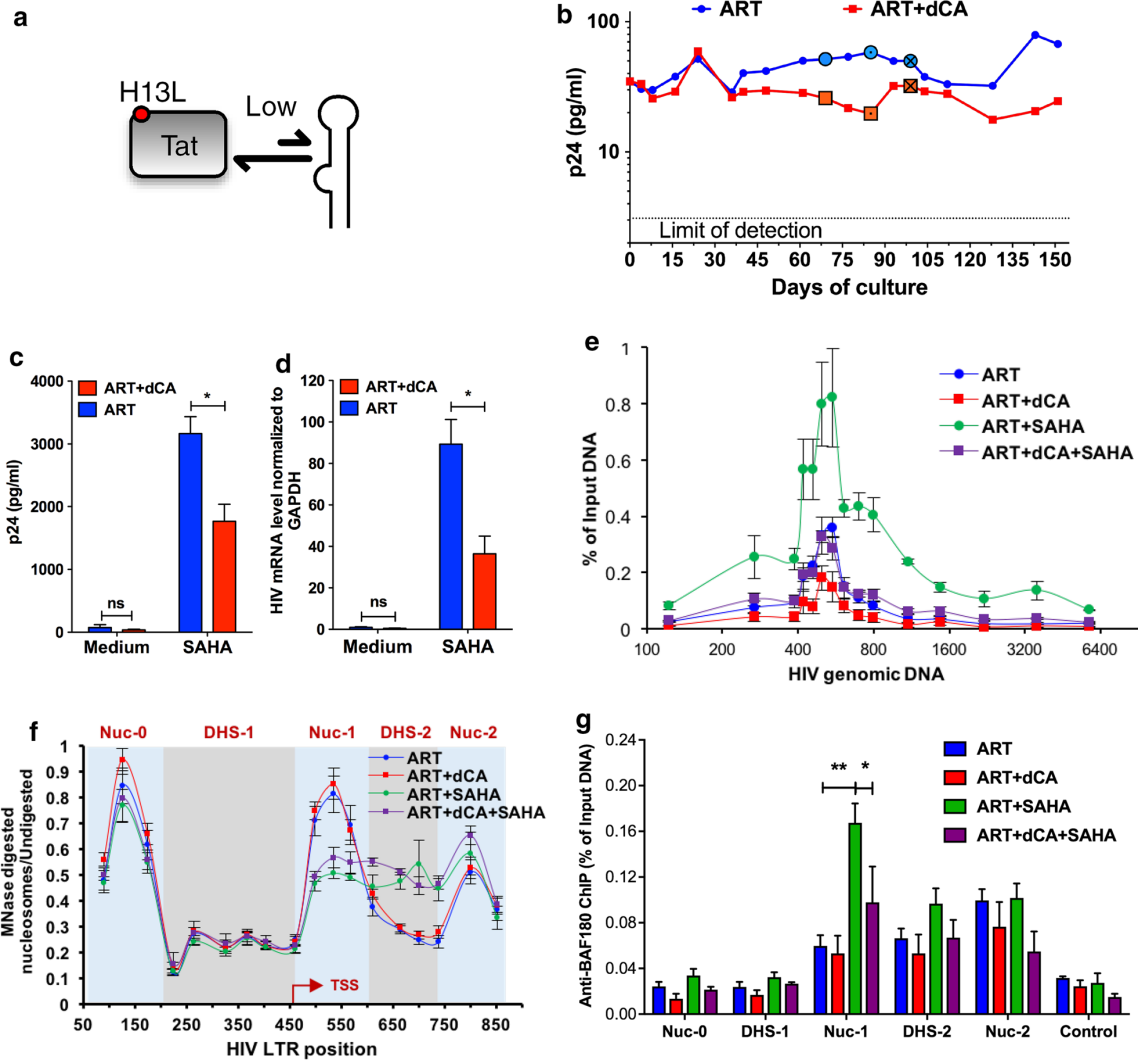
dCA partially inhibits HIV expression in U1 cells. **a** Schematic diagram of the Tat-TAR feedback loop present in provirus in U1 cells. Tat’s H13L mutation weakens interaction with P-TEFb. **b** The viral production in U1 cell treated with dCA. Cells were split and treated on average every 3 days in the presence of ART with or without dCA (10 nM). Capsid production was quantified by p24 ELISA. Data are representative of three independent experiments. **c** The viral production in U1 cell stimulated by SAHA. After 48 days of treatment, cells (highlighted with “⦿/☒” in panel A) were stimulated with 2.5 µM SAHA for 24 h. Capsid production was quantified by p24 ELISA. Data are average of 3 independent experiments and the error bars represent the SD of 3 independent experiments. **d** The viral mRNA levels in U1 cell stimulated by SAHA. Samples from panel B were used for RNA extraction, and cDNAs from extracted total RNA were quantified by RT-PCR using primers to the Vpr region. Results were normalized as the number of viral mRNA copies per GAPDH mRNA. Viral mRNA generated in the ART control was set to 100%, and the error bars represent the SD of 3 independent experiments. **e** Distribution of RNAP II on the HIV genome in U1 cells. ChIP assay to RNAPII was performed using cell samples from panels C-D. Results are represented as the percentage of input and subtracted the background of the isotype IgG control. Data are average of 3 independent experiments, and error bars represent the SD of 3 experiments for each primer set. **f** MNase protective assays using sample in panels B-C. Error bars represent the SD of 3 independent experiments. **g** The recruitment of PBAF complex on HIV promoter DNA in as determined by ChIP to BAF180. Results are presented as percent immunoprecipitated DNA over input after isotype IgG control background subtraction. Data are average of 3 independent experiments, and error bars represent the SD of 3 experiments for each primer set. The promoter of GAPDH was used as the control. Statistical significance was determined using unpaired t-test (ns, no significance, *P < 0.05)

To investigate whether the strength of dCA inhibition of HIV transcription was dependent on the strength of the Tat-TAR feedback loop, we investigated its activity in a latent model characterized by suboptimal Tat-TAR feedback due to attenuated Tat activity (Fig. 3a). In this model, U1, chronically infected clonal cells carry two HIV proviruses, one lacking Tat’s ATG initiation codon, and the other containing Tat’s H13L mutation which weakens its interaction with P-TEFb [58, 59]. Of note, dCA binds to the ARM domain of Tat and thus the H13L mutation in Tat does not affect its interaction with dCA [71]. Long-term treatment of U1 cells with 10 nM of dCA revealed only a modest reduction in viral capsid production after day 36 (Fig. 3b). To investigate dCA’s activity, we stimulated cells treated for more than 48 days with SAHA and measured both capsid production by p24 ELISA (Fig. 3c) and HIV mRNA production by RT-PCR (Fig. 3d). The reactivation of provirus in U1 cells was only partially inhibited by dCA, consistent with the residual transcriptional activity of the H13L Tat [59]. To further explore this moderate inhibitory activity at the chromatin level, we used ChIP to investigate RNAP II recruitment to the HIV promoter and genome. Under non-activated conditions, dCA treatment only moderately reduced RNAP II on the HIV promoter (Fig. 3e, compare blue and red lines), in sharp contrast with what is observed in Fig. 1e with competent Tat-TAR feedback. Since transcription is very low in this latency model, very little elongating RNAPII is detected throughout the HIV genome, even in ART-only-treated cells. SAHA treatment increased by about fourfold RNAP II recruitment to the HIV genome in ART-treated cells, while a twofold RNAP II increase was detected in dCA-treated cells (Fig. 3e, compare green and purple lines). Next, to further investigate nucleosome positioning in this model we performed MNase protection assays as described above. In this U1 latency cell model, under unstimulated conditions, we observed typical latency nucleosomal structure, with the three distinct nucleosomes, and upon SAHA treatment we observed the downstream remodeling of Nuc-1 consistent with transcriptional activation (Fig. 3f). In this model, dCA did not significantly alter the HIV DNA nucleosome occupancy either before or after viral reactivation with SAHA (Fig. 3f). In addition, ChIP to BAF180 (Fig. 3g) did not reveal losses in PBAF recruitment under unstimulated conditions, only after SAHA treatment at the Nuc-1 position. In this U1 model with attenuated Tat activity, dCA only partially blocks viral production upon viral reactivation with SAHA, likely by inhibiting the remaining transcriptional activity of H13L Tat [59]. Taken together, these results suggest that the strength of dCA activity is directly correlated to the strength of the Tat-TAR feedback loop.

### dCA does not inhibit HIV-1 expression in ACH-2 cells with incompetent Tat-TAR activity

**Fig. 4:**
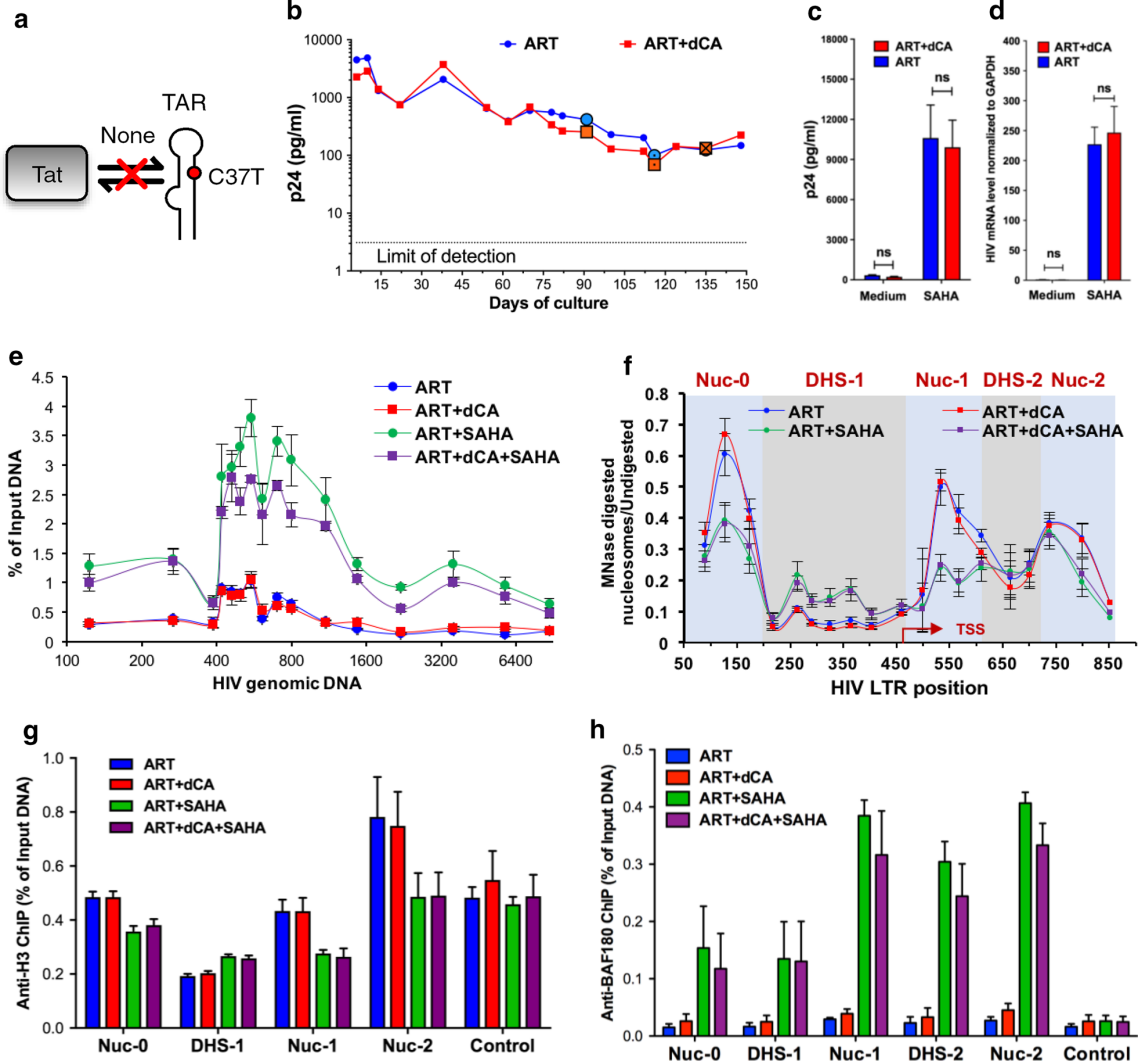
dCA does not inhibit HIV expression in ACH-2 cells. **a** Schematic representation of the Tat-TAR feedback loop present in provirus in ACH-2 cells. **b** The viral production in ACH-2 cell treated with dCA. Cells were split and treated on average every 3 days in the presence of ART with or without 10 nM dCA. Data are representative of three independent experiments. **c** The viral production in ACH-2 cell treated stimulated by SAHA. After 60 days, cells (highlighted with “⦿/☒” in panel A) were stimulated with 2.5 µM SAHA for 24 h. Capsid production was quantified by p24 ELISA. Data are average of 3 independent experiments and the error bars represent the SD of 3 independent experiments. **d** The viral mRNA level in ACH-2 cell treated with SAHA. Samples from panel B were used for RNA extraction, and cDNAs from extracted total RNA were quantified by RT-PCR using primers to the Nef region. Results were normalized as the number of viral mRNA copies per GAPDH mRNA. Viral mRNA generated in the ART control was set to 100%, and the error bars represent the SD of 3 independent experiments. **e** Distribution of RNAP II on the HIV genome in ACH-2 cell. ChIP assay was performed using cells samples from panels B-C. Results are presented as percent immunoprecipitated DNA over input, after isotype IgG control background subtraction. Data are average of 3 independent experiments, and error bars represent the SD of 3 experiments for each primer set. **f** The chromatin structure of the HIV LTR in U1 cells (samples from panel B-C) investigated by MNase protective assays. Error bars represent the SD of 3 independent experiments. **g** The recruitment of PBAF complex on HIV promoter DNA in as determined by ChIP to BAF180. Results are presented as percent immunoprecipitated DNA over input, after isotype IgG control background subtraction. **h** H3 occupancy on HIV promoter DNA determined by ChIP to H3. Results are presented as percent immunoprecipitated DNA over input, after isotype IgG control background subtraction. The ORF of GAPDH was used as the control. Data are average of 3 independent experiments and error bars represent the SD of 3 experiments for each primer set. Statistical significance was determined using unpaired t-test (ns, no significance, *P < 0.05)

**Fig. 5:**
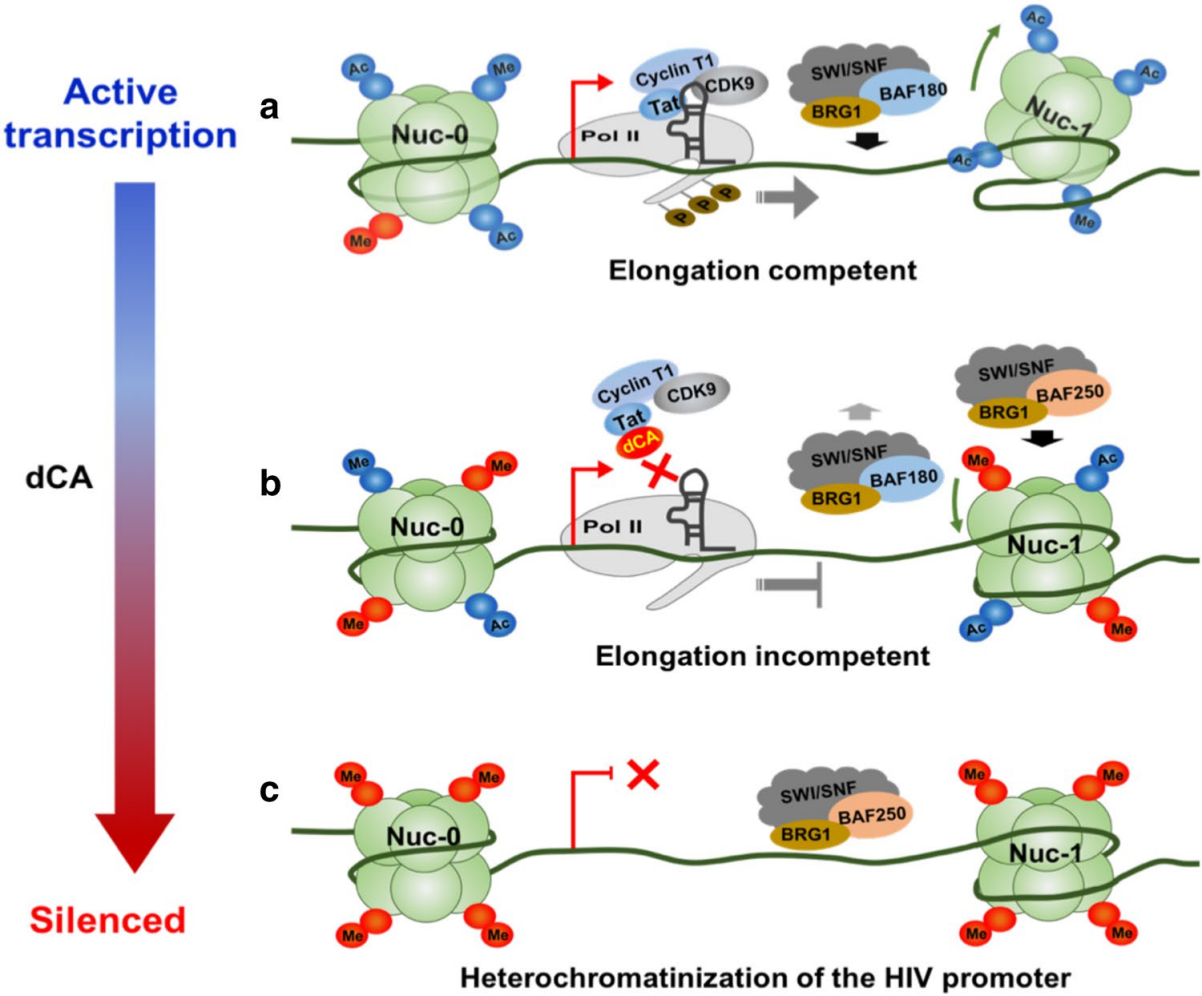
Progressive epigenetic silencing of HIV expression by the TT at inhibitor dCA. **a** During active transcription, Tat recruit P-TEFb to TAR to boost the transition from transcription initiation to elongation. Nuc-1 downstream are characterized with activating epigenetic marks and poised for productive transcription by the recruitment of SWI/SNF chromatin remodeling complex (BAF180). **b** dCA binds to Tat and blocks the recruitment of P-TEFb to TAR, to inhibit the transition of transcription initiation to elongation. The repressive SWI/SNF chromatin remodeling complex (BAF250) accumulates on the HIV promoter, resulting in increased Nuc-1 occupancy and increased repressive epigenetic marks. **c** Overtime the treatment with dCA promotes the establishment of a closed chromatin state with limited RNA polymerase II (RNAP II) recruitment to the promoter. dCA-mediated silencing is refractory to reactivation by various LRAs

To further confirm that dCA inhibits HIV expression and mediates a state of repressive chromatin environment in a Tat-dependent manner, we used the ACH-2 latently infected cell model. These cells contain a provirus which is insensitive to Tat activation due to a C37T mutation in TAR (Fig. 4a) [58, 60]. Despite the very long treatment time with dCA (150 days), we did not observe differences in capsid production between ART- and ART + dCA-treated cells (Fig. 4b). After 60 days in treatment, cells were stimulated with SAHA to assess whether dCA inhibits viral reactivation. As expected, dCA did not block viral reactivation, as determined by p24 ELISA (Fig. 4c) and HIV mRNA analysis (Fig. 4d). Furthermore, ChIP to RNAP II did not reveal differences in RNAP II recruitment to the promoter and genome under basal or SAHA-stimulated condition (Fig. 4e). Moreover, MNase protection assays show the HIV provirus in ACH-2 cell presents the typical latency nucleosomal structure with the three distinct nucleosomes, similar in the presence or absence of dCA, with Nuc-1 remodeling upon SAHA activation reflecting transcriptional activation (Fig. 4f). Similarly, there were no differences in histone occupancy or PBAF recruitment between ART and ART + dCA-treated cells, as determined by ChIP to H3 and BAF180, respectively (Fig. 5g and h). Taken together, these results demonstrate that dCA only promotes a repressive chromatin environment at the HIV promoter DNA, in a Tat-dependent manner through a competent Tat-TAR feedback loop.

## Discussion

Tat is a multitasker protein with the ability to interact with a large number of host proteins to mediate numerous activities [72–74]. For instance, the ARM of Tat (amino acid 49-57) is involved in the interaction of Tat with components of the SWI/SNF complex [16–18, 24], p300 [34, 75], C/EBP [76], Ik-Bα [77], which are involved in HIV latency regulation. The observation that discontinuation of dCA treatment from HIV-1 infected cells did not result in viral rebound (Fig. 1d) and [53, 54], led to speculation that dCA could promote the accumulation of repressive epigenetic changes at the integrated viral promoter. We hypothesized two possible mechanisms by which dCA mediated these epigenetic changes: (1) dCA treatment simply accelerated the establishment of typical proviral latency by facilitating/accelerating the corresponding epigenetic modifications; or (2) established a unique set of epigenetic changes due to the specific block of the subset of Tat functions that was mediated by the ARM [53]. In addition, we seeked to investigate whether dCA activity was directly correlated with Tat-TAR strength. To answer these questions, we exploited HIV-1 cellular models with different transcriptional strengths. Considering the extremely low frequency of latently infected cells in infected individuals on ART (about 1 in 10^6^ CD4^+^ T cells), and the difficulty to conduct biochemical studies using primary cells [78], we opted to use cell lines: HeLa-CD4 cells chronically infected cells with NL43 were used as our model of high transcriptional activity; the promyelocytic OM-10.1 cell line was used as a latent model with low transcriptional activity; U1 cell was used as our model of suboptimal Tat activity; and finally ACH-2 cells as our Tat transcriptional null model.

In HeLa-CD4 and OM-10.1 cells, dCA suppressed residual viral production to an almost undetectable level and blocked the viral production induced by various stimulations (Figs. 1a–c, 2a–c). We also observed that in OM-10.1 cells the inhibition by dCA is detected earlier than that in Hela-CD4 cell (Figs. 1a and 2a). Virus in each cell line may have a specific timing for entrance into deep latency that is probably not just a matter of the initial starting level of p24 production, but also of additional interfering factors such as the cellular activation state, nuclear levels of transcription factors, the site of chromatin integration, or distance from cellular enhancers. In the Hela-CD4 model, not only the viral production was 2 logs higher than in OM-10.1 (about 10^5^ pg/ml versus 10^3^ pg/ml), but also the population of virus is integrated in a variety of chromatin locations. Thus, it may take longer to silence this larger variety of integrated proviruses under different epigenetic contexts, as opposed to OM-10.1 that contain a clonal single integration. Nevertheless, we would like to point out that dCA immediately reduced p24 production in the HeLa CD4 model; however, since the representation is in log-scale it is difficult to observe. We have observed similar results in our previous work [53], in which the p24 levels drop immediately after dCA treatment, flicker a bit and reach the limit of detection at day 82. Interestingly though, dCA was able to shut off transcription in HeLa cells to below the detection limit, but not in OM-10.1 cells. Together our results suggest that dCA efficacy, in a general manner, may be limited by Tat-independent transcriptional activity of the viral promoter, that may depend on whether the provirus is integrated in an active/inactive transcription site [78–80], the site of chromatin integration [81–84], or distance from cellular enhancers [85– 87].

In these two cell models, dCA treatment did not alter the typical nucleosome positioning [12, 88], however induced the deposition of histone marks overtime that lead to a repressive chromatin structure at Nuc-1, rendering viral reactivation much less likely to occur (Figs. 1 and 2). ChIP assays also confirmed lower levels of acetylated H3K27 at Nuc-1 positioning, which marks transcriptional activation by loosening the interaction of H3 with DNA (Figs. 1 and 2). The PBAF complex, key for Nuc-1 remodeling and Tat-activated transcription [89], was significantly reduced in both cell models while BAF was enriched (Figs. 1i and 2h). All these changes are hallmarks of heterochromatin formation. The U1 HIV latency model, reflecting a provirus with suboptimal Tat activity (Tat mutation H13L), revealed an epigenetic signature for dCA-treated cells very similar to ART-only-treated cells, except after viral reactivation with an LRA. The nucleosomal protection of the proviral DNA was identical in ART- or ART + dCA-treated cells, with identical downstream remodeling during SAHA treatment consistent with transcriptional elongation. The only differences observed as a result of dCA treatment were reduced RNAPII and PBAF recruitment upon SAHA activation (Fig. 3e and g). The activity of dCA in this model is consistent with the poor ability of H13L Tat to recruit P-TEFb to TAR [59]. In ACH-2 cells, the provirus carries a C37T mutation in TAR that precludes Tat binding. As such, dCA was completely inactive in this model. dCA did not mediate changes in nucleosomal/DNA association (Fig. 4f), PBAF complex (Fig. 4g) and RNAP II recruitment to the promoter (Fig. 4e), nor viral production (Fig. 4c and d).

In Sum, our results using these four cell line models of HIV-1 transcription consistently point to a mechanism where dCA treatment simply accelerates the establishment of typical proviral latency. Furthermore, they clearly suggest that dCA activity is directly correlated with Tat-TAR competence. Specifically, we did not observe epigenetic changes in other unrelated promoters tested (Figs. 1 and 2) suggesting limited off target effects, nor in HIV-1 proviruses in which Tat and TAR were not communicating (Fig. 4). In sum, dCA binds to Tat and blocks Tat-TAR interaction to suppress transcription elongation, and as transcriptional events become increasingly scarce, repressive chromatin marks are progressively deposited at the HIV-1 promoter chromatin, especially Nuc-1, eventually driving the provirus into profound latency refractory to viral reactivation (Fig. 5) [90–92].

This ability of dCA to promote heterochromatinization of the HIV-1 promoter to suppress viral reactivation, further highlights the key role of Tat in the regulation of HIV activation and latency [48–51]. The addition of a Tat inhibitor such as dCA to an ART-regimen could promote and maintain a state of latency, possibly allowing for ART interruption without viral rebound. With time, patients may observe a reduction in the size of the viral reservoir through inhibition of potential reservoir replenishment events and relief from chronic inflammation caused by ongoing low level of virus production [53, 93, 94]. Collectively our study explains the potent inhibitory effect of dCA on HIV-1 infection at the epigenetic level, highlighting the advantage of the introducing Tat inhibitor in “block-and-lock” eradication strategies.

## Additional files

**Additional file 1: Table 1.** Sequences of primers used.

**Additional file 2: Figure S1.** dCA inhibits HIV expression in HIV chronically infected HeLa-CD4 cells. **a** HIV integration level in the chronically infected HeLa-CD4 cell model. Genomic DNA extracted on the indicated days of dCA treatment was amplified by Alu-PCR followed by a nested RT-PCR. HIV proviruses were normalized to genomic GAPDH DNA. Data are the mean ± standard error. **b** Induction of TBP-2 expression by SAHA. The chronically infected cells grown more than 280 days in dCA and fresh HeLa-CD4 cell were activated with SAHA for 24 h. TBP-2 mRNA production was quantified from cDNAs prepared from total RNA. Results were normalized as mRNA copies per GAPDH mRNA, and data represent mean ± standard error. Results are representative of two independent experiments. **c** Induction of IL-1β expression by PMA/Ionomycin in the long-term-treated HeLa-CD4 cells. Cells grown more than 280 days and fresh HeLa-CD4 cells were activated with PMA/ionomycin for 24 h. IL-1β mRNA production was quantified from cDNAs prepared from total RNA. Results were normalized as mRNA copies per GAPDH mRNA, and data represent the mean ± standard error. Results are representative of two independent experiments. **d** Induction of histone H3 acetylation by SAHA in long-term-treated HeLa-CD4 cell. The cell samples from panel B were used for WB analysis with antibody recognizing total histone H3 or N-terminus acetylated H3. The amount of acetylated-H3 was normalized to total histone H3 and labeled below. Results are representative of two independent experiments. **e** PMA/ionomycin-induced viral production in HeLa-CD4 chronic infected cells. Cells treated with ART and ART + dCA (10 nM) after day 280 were stimulated with PMA/Ionomycin for 24 h. Capsid production was quantified via a p24 ELISA. Data are average of 3 independent experiments, and the error bars represent the SD of 3 independent experiments (ND, not detected). **f** PMA/ionomycin-induced viral mRNAs production in HeLa-CD4 chronic infected cells. Cellular samples from panel B were used for RNA extraction, and cDNAs from extracted total RNA were quantified by RT-qPCR using primers to the Nef region. Results were normalized as the number of viral mRNA copies per GAPDH mRNA. Viral mRNA generated in the ART control was set to 100%, and the error bars represent the SD of 3 independent experiments. **g** Distribution of RNAPII on the HIV genome treated or not with dCA. ChIP assay was performed on cells samples from panels F and G. After subtracted with the background of the isotype IgG control, the results are presented as percent immunoprecipitated DNA over input. Error bars represent the SD of 3 experiments for each primer set. **h** The chromatin structure of the HIV LTR in chronic infected HeLa-CD4 cell stimulated with or without PMA/ionomycin. Data are average of 3 independent experiments, and error bars represent the SD of 3 experiments for each primer set. **i** The recruitment of PBAF complex on HIV promoter DNA in cells stimulated with PMA/Ionomycin as determined by BAF180 ChIP. After subtracted with the background of the isotype IgG control, the results are presented as percent immunoprecipitated DNA over input. The promoter of GAPDH was used as the control. Data are average of 3 independent experiments, and error bars represent the SD of 3 experiments for each primer set. **j** The recruitment of BAF complex on HIV promoter DNA in cells stimulated with PMA/Ionomycin as determined by BAF250 ChIP. After subtracted with the background of the isotype IgG control, the results are presented as percent immunoprecipitated DNA over input. The promoter of GAPDH was used as the control. Data are average of 3 independent experiments, and error bars represent the SD of 3 experiments for each primer set. Statistical significance was determined using the unpaired t-test (*P < 0.05, **P < 0.01).
