# Supplementary material for: Tat inhibition by didehydro-Cortistatin A promotes heterochromatin formation at the HIV-1 long terminal repeat
Source: Epigenetics Chromatin. 2019 Apr 16;12:23. doi: 10.1186/s13072-019-0267-8 (PMC6466689; doi:10.1186/s13072-019-0267-8)
Supplement: Supplementary file 1 — Additional file 1: Table 1. Sequences of primers used. [file 13072_2019_267_MOESM1_ESM.pdf]

Primer pairs used for MNase experiments Real-time-PCR (RT-PCR) in Hela-CD4 cell:

p1-F	GATCTGTGGATCTACCACAC
p1-R	GCACCATCCAAAGGTCAGTGG
p2-F	CCTGATTGGCAGAACTACACAC
p2-R	TCTACTTGCTCTGGTTCAACTGG
p3-F	CCTTTGGATGGTGCTTCAAGTTAG
p3-R	ATGCTGGCTCATAGGGTGTAAC3
p4-F	GAGCAAGTAGAAGAGGCCAATG
p4-R	GAAATGCTAGGAGGCTGTCA
p5-F	GAGCCAGCATGGGATGG
p5-R	CTCCGGATGCAGCTCTC
p6-F	CCCGGAGGGAGAAGTATTAGTGT
p6-R	CCCTTGTAGAAAGCTCGATGTCA
p7-F	TGACAGCCTCCTAGCATTTC
p7-R	CACACCTCCCTGGAAAGTC3
p8-F	TACTACAAAGACTGCTGACATCG
p8-R	TCTGAGGGCTCGCCACTC
p9-F	TCTACAAGGGACTTTCCGCTGG
p9-R	GCTTATATGTAGCATCTGAGGGCT
p10-F	CTTTCCAGGGAGGTGTGGCCT
p10-R	GGTCTAACCAGAGAGACCCAGTA
p11-F	AGTGGCGAGCCCTCAGATG
p11-R	AGCAGTGGGTTCCCTAGTTAGC
p12-F	CCTGTACTGGGTCTCTCTGGTT
p12-R	TTTGAGCACTCAAGGCAAGCTTTA
p13-F	CTGGGAGCTCTCTGGCTAACTA
p12-R	TTACCAGAGTCACACAACAGACG
p14-F	CCACTGCTTAAGCCTCAATAAAGCT
p14-R	TCCACACTGACTAAAAGGGTCTGA
p15-F	AGTGTGTGCCCGTCTGTTGTG
p15-R	CTTTCGCTTTCAAGTCCCTGTTCG
p16-F	GTGTGGAAAATCTCTAGCAGTG
p16-R	CTTCAGCAAGCCGAGTCC
p17-F	CGAACAGGGACTTGAAAGCGAAA
p17-R	CGTACTCACCAGTCGCCGC
p18-F	GAGATCTCTCGACGCAGGACT
p18-R	CGCACCCATCTCTCTCCTTCTA
p19-F	GCGACTGGTGAGTACGCCAA
p19-R	CCCCTGGCCTTAACCGAATTT
p20-F	GGTGCGAGAGCGTCGGTATT
P20-R	GTTCCCTGCTTGCCCATACT

Primer pairs used for MNase experiments RT-PCR in U1 cell:

p1-F	GATCTGTGGATCTACCACAC
p1-R	GCACCATCCAAAGGTCAGTGG
p2-F	CCCTGATTGGCAGAACTACACAC
p2-R	CCTTTCTACCTGCCCTGGCT
p3-F	CCTTTGGATGGTGCTTCAAGTTAG
p3-R	ATGCAGGCTCATAGGGTGTAAC
p4-F	GGGCAGGTAGAAAGGGCCA
p4-R	GAAATGCTAGGAGGCTGTCA
p5-F	GAGCCTGCATGGGATGG
p5-R	CTCCGGATGCAGCTCTC
p6-F	TGACAGCCTCCTAGCATTTC
p6-R	CACACCTCCCTGGAAAGTC3
p7-F	TACTACAAAGACTGCTGACATCG
p7-R	TCTGAGGGCTCGCCACTC
p8-F	GGGACTTTCCGCTGGGGAC
p8-R	CCCAGTACAGGCAAAAAGCAGC
p9-F	AGTGGCGAGCCCTCAGATG
p9-R	AAGCAGTGGGTTCCCTAGCTA
p10-F	CCTGTACTGGGTCTCTCTGGTT
p10-R	TTGTAGCACTCAAGGCAAGCTTTA
p11-F	CTGGGAGCTCTCTGGCTAGCTA
p11-R	TTACCAGAGTCACACAACAGACG
p12-F	CCACTGCTTAAGCCTCAATAAAGCT
p12-R	TCCACACTGACTAAAAGGGTCTGA
p13-F	AGTGTGTGCCCGTCTGTTGTG
p12-R	CTTTCGCTTTCAAGTCCCTGTTCG
p14-F	GTGTGGAAAATCTCTAGCAGTG
p14-R	CTTCAGCAAGCCGAGTCC
p15-F	CGAACAGGGACTTGAAAGCGAAA
p15-R	TTTGGCGTACTCACCAGTCGC
p16-F	GAGATCTCTCGACGCAGGACT
p16-R	CGCACCCATCTCTCTCCTTCTA
p17-F	GCGACTGGTGAGTACGCCAA
p17-R	CCCCTGGCCTTAACCGAATTT
p18-F	GGTGCGAGAGCGTCGGTATT
p18-R	GTTCCCTGCTTGCCCATACT

Primer pairs used for MNase experiments RT-PCR in ACH2 cell:

p1-F	GATCTGTGGATCTACCACAC
p1-R	GCACCATCCAAAGGTCAGTGG
p2-F	CCCTGATTGGCAGAACTACACAC
p2-R	GGCCTCTTCTACCTTATCTGGCT
p3-F	CCTTTGGATGGTGCTACAAGCTAG
p3-R	ATGCAGGCTCACAGGGTGTAAC
p4-F	GCCAGATAAGGTAGAAGAGGCCAA
p4-R	GCGGCTGTCAAACCTCCACT
p5-F	GTGAGCCTGCATGGAATGGAT
p5-R	CTCCGGATGCAGCTCTC
p6-F	TGACAGCCGCCTAGCATTTCA
p6-R	CACGCCTCCCTGGAAAGTC
p7-F	GGAGTACTTCAAGAACTGCTGACA
p7-R	TCTGAGGGCTCGCCACTC
p8-F	GGGACTTTCCGCTGGGGAC
p8-R	CCCAGTACAGGCAAAAAGCAGC
p9-F	AGTGGCGAGCCCTCAGATG
p9-R	AGCAGTGGGTTCCCTAGTTAGC
p10-F	CCTGTACTGGGTCTCTCTGGTT
p10-R	TTGAAGCACTCAAGGCAAGCTTTA
p11-F	GCTGGGAGTTCTCTGGCTAACTA
p11-R	CAGAGTCATACAACAGACGGGCA
p12-F	CCACTGCTTAAGCCTCAATAAAGCT
p12-R	TCCACACTGACTAAAAGGGTCTGA
p13-F	AGTGTGTGCCCGTCTGTTGTA
p12-R	CTTTCGCTTTCAGGTCCCTGTTC
p14-F	GTGTGGAAAATCTCTAGCAGTG
p14-R	CTTCAGCAAGCCGAGTCC
p15-F	CGAACAGGGACCTGAAAGCGAA
p15-R	TTTGGCGTACTCACCAGTCGC
p16-F	GAGCTCTCTCGACGCAGGACT
p16-R	CGCACCCATCTCTCTCCTTCTA
p17-F	GCGACTGGTGAGTACGCCAA
p17-R	CCCCTGGCCTTAACCGAATTT
p18-F	GGTGCGAGAGCGTCAGTATT
p18-R	GCTCCCTGCTTGCCCATACT

Primer pairs used for RNAPII ChIP experiments RT-PCR in Hela-CD4 cell:

p1-F	CCCTGATTGGCAGAACTACACAC
p1-R	TCTACCTTATCTGGCTCAACTGGT
p2-F	GAGCCTGCATGGAATGGATGA
p2-R	GAAGTACTCCGGATGCAGCTCT
p3-F	GCTACAAGGGACTTTCCGCT
p3-R	GCTTATATGCAGCATCTGAGGGCT
p4-F	GGACTTTCCAGGGAGGCGT
p4-R	GGTCTAACCAGAGAGACCCAGTA
p5-F	AGTGGCGAGCCCTCAGATG
p5-R	AGCAGTGGGTTCCCTAGTTAGC
p6-F	CCTGTACTGGGTCTCTCTGGTT
p6-R	TTTGAGCACTCAAGGCAAGCTTTA
p7-F	TCTCTGGCTAACTAGGGAACC
p7-R	AAAGGGTCTGAGGGATCTCTAG
p8-F	AGTGTGTGCCCGTCTGTTGTG
p8-R	CTTTCGCTTTCAAGTCCCTGTTCG
p9-F	CGAACAGGGACTTGAAAGCGAAA
p9-R	CGTACTCACCAGTCGCCGC
p10-F	GCGACTGGTGAGTACGCCAA
p10-R	CCCCTGGCCTTAACCGAATTT
p11-F	GCAGTCCTCTATTGTGTGCATCAA
p11-R	CCTGTGTCAGCTGCTGCTTG
p12-F	CCATCAATGAGGAAGCTGCAGAA
p12-R	GGTGGATTATGTGTCATCCATCCT
p13-F	TTCTTCAGAGCAGACCAGAGC
p12-R	GCTGCCAAAGAGTGATCTGA
p14-F	CAGAAATACAGAAGCAGGGGCAA
p14-R	GTGTGGGCACCCTTCATTCTT
p15-F	ACTTACGGGGATACTTGGGCAG
p15-R	CTCCATTTCTTGCTCTCCTCTGTC
p16-F	TTGCTCAATGCCACAGCCAT
p16-R	TTTGACCACTTGCCACCCAT

Primer pairs used for RNAPII ChIP experiments RT-PCR in U1 cell:

p1-F	CCCTGATTGGCAGAACTACACAC
p1-R	CCTTTCTACCTGCCCTGGCT
p2-F	GAGCCTGCATGGGATGG
p2-R	CTCCGGATGCAGCTCTC
p3-F	TCTACAAGGGACTTTCCGCTGG

p3-R	GCTTATATGTAGCATCTGAGGGCT
p4-F	CTTTCCAGGGAGGTGTGGCCT
p4-R	GGTCTAACCAGAGAGACCCAGTA
p5-F	AGTGGCGAGCCCTCAGATG
p5-R	AAGCAGTGGGTTCCCTAGCTA
p6-F	CCTGTACTGGGTCTCTCTGGTT
p6-R	TTGTAGCACTCAAGGCAAGCTTTA
p7-F	CTGGGAGCTCTCTGGCTAGCTA
p7-R	TTACCAGAGTCACACAACAGACG
p8-F	AGTGTGTGCCCGTCTGTTGTG
p8-R	CTTTCGCTTTCAAGTCCCTGTTCG
p9-F	CGAACAGGGACTTGAAAGCGAAA
p9-R	TTTGGCGTACTCACCAGTCGC
p10-F	GCGACTGGTGAGTACGCCAA
p10-R	CCCCTGGCCTTAACCGAATTT
p11-F	GCAGTCCTCTATTGTGTGCATCAA
p11-R	CCTGTGTCAGCTGCTGCTTG
p12-F	CCATCAATGAGGAAGCTGCAGAA
p12-R	GGTGGATTATGTGTCATCCATCCT
p13-F	TTCTTCAGAGCAGACCAGAGC
p12-R	GCTGCCAAAGAGTGATCTGA
p14-F	CAGAAATACAGAAGCAGGGGCAA
p14-R	GTGTGGGCACCCTTCATTCTT
p15-F	ACTTACGGGGATACTTGGGCAG
p15-R	CTCCATTTCTTGCTCTCCTCTGTC

Primer pairs used for RNAPII ChIP experiments RT-PCR in ACH2 cell:

p1-F	CCCTGATTGGCAGAACTACACAC
p1-R	GGCCTCTTCTACCTTATCTGGCT
p2-F	GTGAGCCTGCATGGAATGGAT
p2-R	CTCCGGATGCAGCTCTC
p3-F	TCTACAAGGGACTTTCCGCTGG
p3-R	GCTTATATGTAGCATCTGAGGGCT
p4-F	CTTTCCAGGGAGGTGTGGCCT
p4-R	GGTCTAACCAGAGAGACCCAGTA
p5-F	AGTGGCGAGCCCTCAGATG
p5-R	AGCAGTGGGTTCCCTAGTTAGC
p6-F	CCTGTACTGGGTCTCTCTGGTT
p6-R	TTGAAGCACTCAAGGCAAGCTTTA
p7-F	GCTGGGAGTTCTCTGGCTAACTA

p7-R	CAGAGTCATACAACAGACGGGCA
p8-F	AGTGTGTGCCCGTCTGTTGTA
p8-R	CTTTCGCTTTCAGGTCCCTGTTC
p9-F	CGAACAGGGACCTGAAAGCGAA
p9-R	TTTGGCGTACTCACCAGTCGC
p10-F	GCGACTGGTGAGTACGCCAA
p10-R	CCCCTGGCCTTAACCGAATTT
p11-F	GCAGTCCTCTATTGTGTGCATCAA
p11-R	CCTGTGTCAGCTGCTGCTTG
p12-F	CCATCAATGAGGAAGCTGCAGAA
p12-R	GGTGGATTATGTGTCATCCATCCT
p13-F	TTCTTCAGAGCAGACCAGAGC
p12-R	GCTGCCAAAGAGTGATCTGA
p14-F	CAGAAATACAGAAGCAGGGGCAA
p14-R	GTGTGGGCACCCTTCATTCTT
p15-F	ACTTACGGGGATACTTGGGCAG
p15-R	CTCCATTTCTTGCTCTCCTCTGTC
p16-F	TTGCTCAATGCCACAGCCAT
p16-R	TTTGACCACTTGCCACCCAT

Primer pairs used for Histone and BAF ChIP experiments RT-PCR in Hela-CD4 and OM10.1 cell:

Nuc-0-F	CCTGATTGGCAGAACTACACAC
Nuc-0-R	TCTACTTGCTCTGGTTCAACTGG
DHS-1-F	TGACAGCCTCCTAGCATTTC
DHS-1-R	CACACCTCCCTGGAAAGTC3
Nuc-1-F	CTGGGAGCTCTCTGGCTAACTA
Nuc-1-R	TTACCAGAGTCACACAACAGACG
DHS-2-F	GAAAGCGAAAGTAAAGCCAGAGGA
DHS-2-R	TTTGGCGTACTCACCAGTCGC
Nuc-2-F	GCGACTGGTGAGTACGCCAA
Nuc-2-R	CCCCTGGCCTTAACCGAATTT

Primer pairs used for Histone and BAF ChIP experiments RT-PCR in U1 cell:

Nuc-0-F	CCCTGATTGGCAGAACTACACAC
Nuc-0-R	CCTTTCTACCTGCCCTGGCT
DHS-1-F	TGACAGCCTCCTAGCATTTC
DHS-1-R	CACACCTCCCTGGAAAGTC3
Nuc-1-F	CTGGGAGCTCTCTGGCTAGCTA
Nuc-1-R	TTACCAGAGTCACACAACAGACG
DHS-2-F	CGAACAGGGACTTGAAAGCGAAA
DHS-2-R	TTTGGCGTACTCACCAGTCGC

Nuc-2-F	GCGACTGGTGAGTACGCCAA
Nuc-2-R	CCCCTGGCCTTAACCGAATTT

Primer pairs used for Histone and BAF ChIP experiments RT-PCR in ACH2 cell:

Nuc-0-F	CCCTGATTGGCAGAACTACACAC
Nuc-0-R	GGCCTCTTCTACCTTATCTGGCT
DHS-1-F	TGACAGCCGCCTAGCATTTCA
DHS-1-R	CACGCCTCCCTGGAAAGTC
Nuc-1-F	GCTGGGAGTTCTCTGGCTAACTA
Nuc-1-R	CAGAGTCATACAACAGACGGGCA
DHS-2-F	GTGTGGAAAATCTCTAGCAGTG
DHS-2-R	CTTCAGCAAGCCGAGTCC
Nuc-2-F	GAGCTCTCTCGACGCAGGACT
Nuc-2-R	CGCACCCATCTCTCTCCTTCTA

Control primer pairs used for Histone and BAF ChIP experiments RT-PCR:

GAPDH-F	GGACCTGACCTGCCGTCTAGAA
GAPDH-R	GGTGTCGCTGTTGAAGTCAGAG
RPL-10-F	ACCCGTCTTCGACAGGACT
RPL-10-R	GGAACGGAAGACGAGAACAG

Primer pairs used for mRNA and Alu-PCR analysis:

HIV-mRNA-F	TTGCTCAATGCCACAGCCAT
HIV-mRNA-R	TTTGACCACTTGCCACCCAT
GAPDH-mRNA-F	CAACAGCCTCAAGATCATCAGCA
GAPDH-mRNA-R	AGGGATGACCTTGCCCACAGCCTTGG
TBP2-mRNA-F	TGTGTGAAGTTACTCGTGTCAAA
TBP2-mRNA-R	GCAGGTACTCCGAAGTCTGT
IL-1b-mRNA-F	GTGGCAATGAGGATGACTTGTTC
IL-1b-mRNA-R	TAGTGGTGGTCGGAGATTCGTA
Alu1-F	TCCCAGCTACTGGGGAGGCTGAGG
Gag-R	CCTGTGTCAGCTGCTGCTTG
HIV-gag-F	GGCTAACTAGGGAACCCACTG
HIV-gag-R	CTGCTAGAGATTTTCCACACTGAC
