# Supplementary figures and images for: Tat inhibition by didehydro-Cortistatin A promotes heterochromatin formation at the HIV-1 long terminal repeat
Source: Epigenetics Chromatin. 2019 Apr 16;12:23. doi: 10.1186/s13072-019-0267-8 (PMC6466689; doi:10.1186/s13072-019-0267-8)

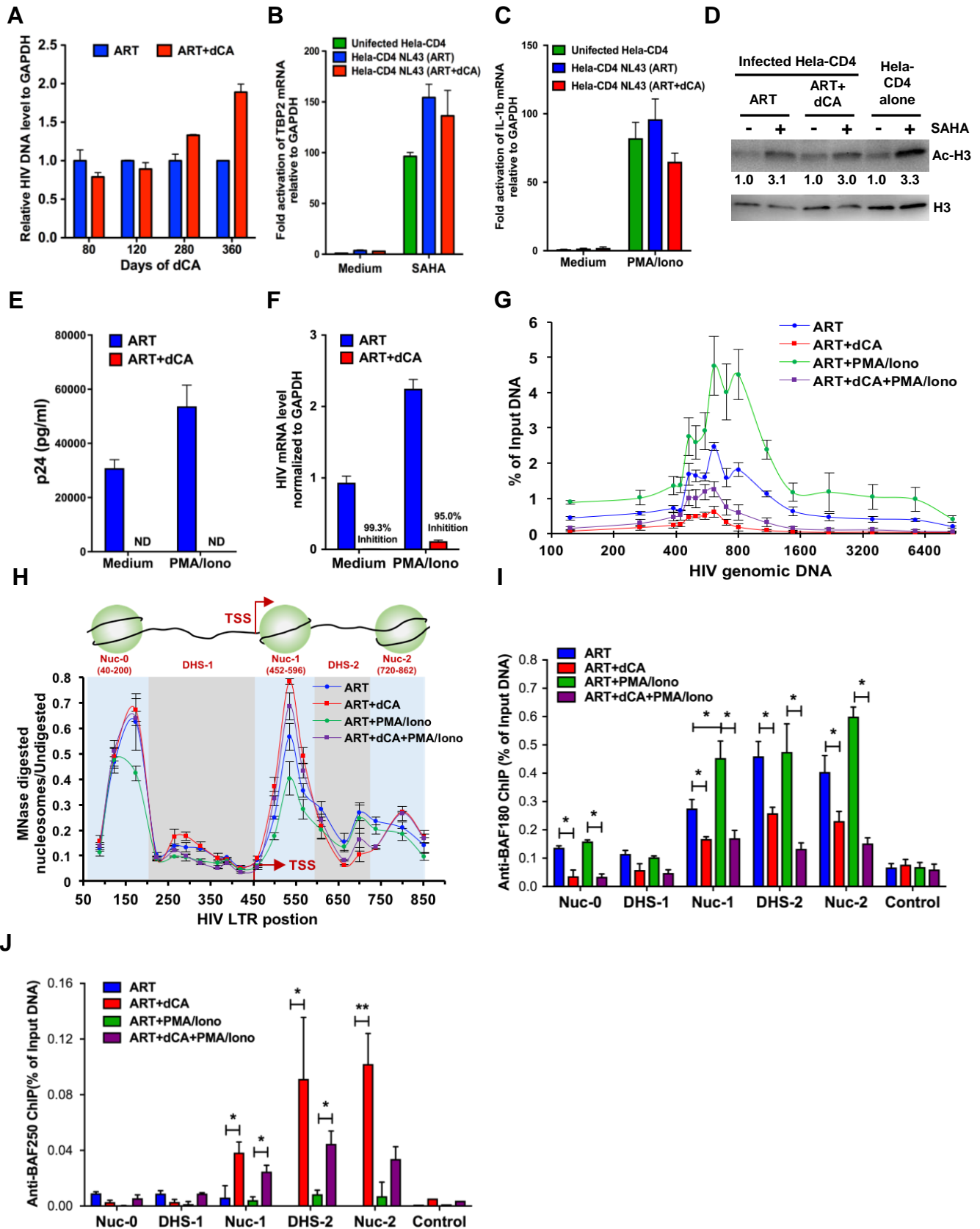

Supplement: Supplementary file 2 — Additional file 2: Figure S1. dCA inhibits HIV expression in HIV chronically infected HeLa-CD4 cells. a HIV integration level in the chronically infected HeLa-CD4 cell model. Genomic DNA extracted on the indicated days of dCA treatment was amplified by Alu-PCR followed by a nested RT-PCR. HIV proviruses were normalized to genomic GAPDH DNA. Data are the mean ± standard error. b Induction of TBP-2 expression by SAHA. The chronically infected cells grown more than 280 days in dCA and fresh HeLa-CD4 cell were activated with SAHA for 24 h. TBP-2 mRNA production was quantified from cDNAs prepared from total RNA. Results were normalized as mRNA copies per GAPDH mRNA, and data represent mean ± standard error. Results are representative of two independent experiments. c Induction of IL-1β expression by PMA/Ionomycin in the long-term-treated HeLa-CD4 cells. Cells grown more than 280 days and fresh HeLa-CD4 cells were activated with PMA/ionomycin for 24 h. IL-1β mRNA production was quantified from cDNAs prepared from total RNA. Results were normalized as mRNA copies per GAPDH mRNA, and data represent the mean ± standard error. Results are representative of two independent experiments. d Induction of histone H3 acetylation by SAHA in long-term-treated HeLa-CD4 cell. The cell samples from panel B were used for WB analysis with antibody recognizing total histone H3 or N-terminus acetylated H3. The amount of acetylated-H3 was normalized to total histone H3 and labeled below. Results are representative of two independent experiments. e PMA/ionomycin-induced viral production in HeLa-CD4 chronic infected cells. Cells treated with ART and ART + dCA (10 nM) after day 280 were stimulated with PMA/Ionomycin for 24 h. Capsid production was quantified via a p24 ELISA. Data are average of 3 independent experiments, and the error bars represent the SD of 3 independent experiments (ND, not detected). f PMA/ionomycin-induced viral mRNAs production in HeLa-CD4 chronic infected cells. Cellular [file 13072_2019_267_MOESM2_ESM.pdf]
